# Supplementary material for: Fumarate induces vesicular release of mtDNA to drive innate immunity
Source: Nature. 2023 Mar 8;615(7952):499–506. doi: 10.1038/s41586-023-05770-w (PMC10017517; doi:10.1038/s41586-023-05770-w)
Supplement: Supplementary file 1 — Supplementary Figs. 1–10, including the uncropped Immunoblots (shown as Supplementary Fig. 10) and Supplementary Tables 4 and 5. [file 41586_2023_5770_MOESM1_ESM.pdf]

nature portfolio

https://doi.org/10.1038/s41586-023-05770-w

Supplementary information

Fumarate induces vesicular release of mtDNA to drive innate immunity

In the format provided by the
authors and unedited

Fumarate induces vesicular release of mtDNA to drive innate immunity

Vincent Zecchini[1,16], Vincent Paupe[2,16], Irene Herranz-Montoya[1,8], Joelle Janssen[1,9], Inge M.N. Wortel[1,10], Jordan L. Morris[2], Ashley Ferguson[1], Suvagata Roy Chowdury[2], Marc Segarra-Mondejar[1,11], Ana S. H. Costa[1,12], Goncalo C. Pereira[2], Laura Tronci[1,13], Tim Young[1], Efterpi Nikitopoulou[1], Ming Yang[1,11], Dóra Bihary[1,14], Federico Caicci[3], Shun Nagashima[2,15], Alyson Speed[1], Kalliopi Bokea[4], Zara Baig[5], Shamith Samarajiwa[1], Maxine Tran[4], Thomas Mitchell[6,7], Mark Johnson[2], Julien Prudent[2,17,*] & Christian Frezza[1,11,17,*]

[1]Medical Research Council Cancer Unit, University of Cambridge, Cambridge, UK.

[2]Medical Research Council Mitochondrial Biology Unit, University of Cambridge, Cambridge, UK.

[3]Department of Biology, University of Padova, Padova Italy.

[4]Department of Surgical Biotechnology, Division of Surgery and Interventional Science, UCL, London, UK.

[5]Division of Infection and Immunity, Institute of Immunity and Transplantation, UCL, London, UK.

[6]Wellcome Sanger Institute, Wellcome Genome Campus, Hinxton, Cambridge, UK.

[7]Department of Surgery, University of Cambridge, Cambridge, UK.

[8]Present address: Molecular Oncology Programme, Growth Factors, Nutrients and Cancer Group, Centro Nacional de Investigaciones Oncológicas (CNIO), Madrid, Spain.

[9]Present address: Human and Animal Physiology, Wageningen University and Research, Wageningen, the Netherlands.

[10]Present address: Department of Data Science, institute for Computing and information Sciences, Radboud University, Nijmegen, the Netherlands.

[11]Present address: CECAD Research Centre, University of Cologne, Cologne, Germany.

[12]Present address: Matterworks, Somerville, MA, USA.

[13]Present address: Cogentech SRL Benefit Corporation, Milan, Italy.

[14]Present address: VIB KU Leuven Center for Cancer Biology, Leuven, Belgium.

[15]Present address: Laboratory of Regenerative Medicine, School of Life Sciences, Tokyo University of Pharmacy and Life Sciences, Tokyo, Japan.

[16]These authors contributed equally: Vincent Zecchini, Vincent Paupe.

[17]These authors jointly supervised this work: Julien Prudent, Christian Frezza.

[*]e-mail: julien.prudent@mrc-mbu.cam.ac.uk; christian.frezza@uni-koeln.de

This Supplementary information file contains:

- Supplementary Figures S1- S10
- Supplementary Tables 1-3 legends, and Supplementary Tables 4-5

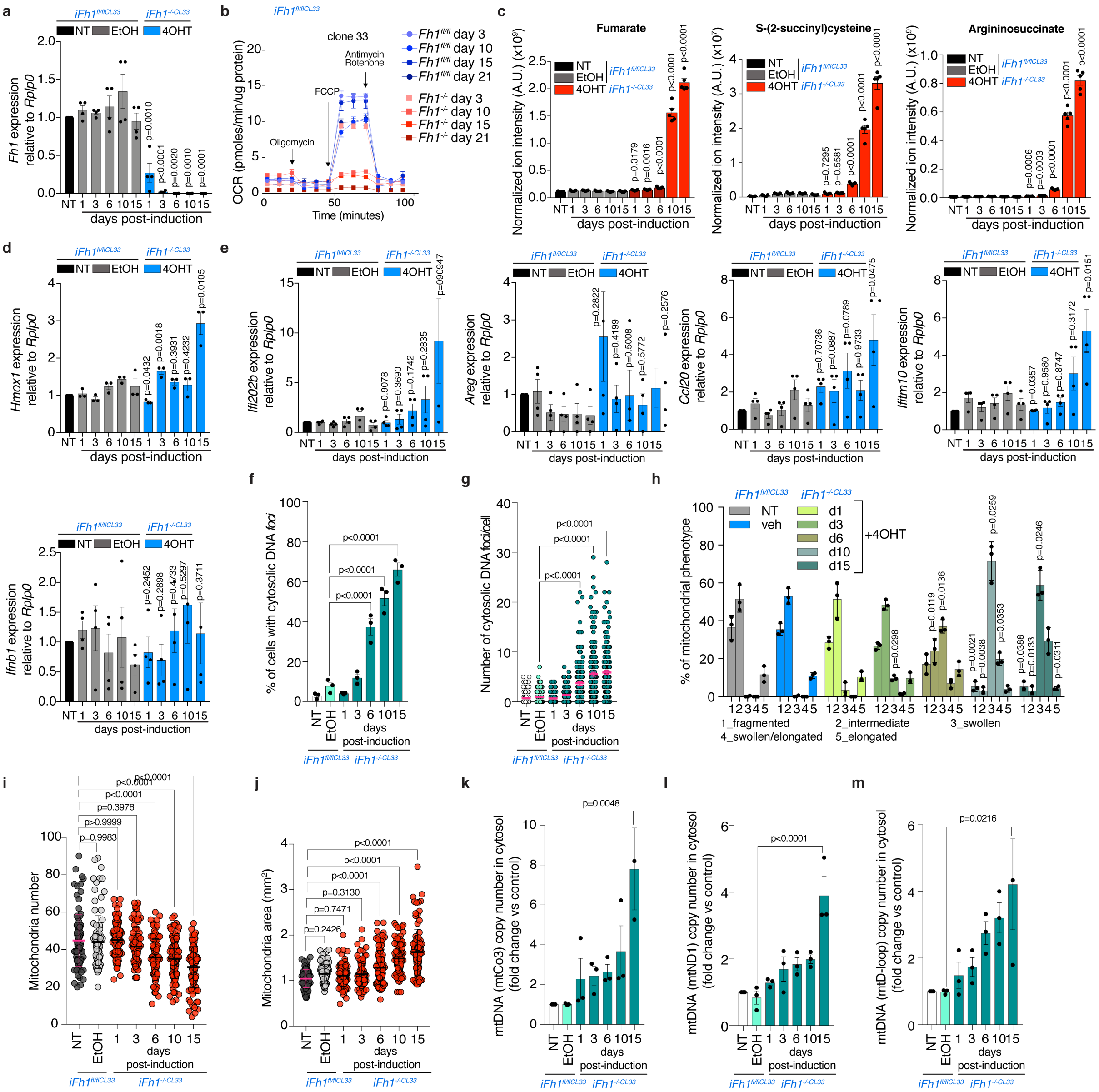

Supplementary Figure 1

a, qRT-PCR showing expression levels of *Fh1* in inducible *iFh1* epithelial kidney cell lines clones 33 (*iFh1*$^{fl/flC33}$) untreated (NT) or treated with either vehicle (ethanol; EtOH) or 4-hydroxytamoxifen (4-OHT) (*iFh*$^{-/-CL33}$) for the indicated period of time. *n* = 5 independent experiments. **b**, Mitochondrial respiration measured using Seahorse in *iFh*^{CL33} cells. *n* = 3 independent experiments. **c**, Relative abundance of fumarate (left), S-(2-succinyl)cysteine (middle) and argininosuccinate (right) in *iFh1*^{CL33} cells measured by liquid-chromatography mass-spectrometry (LC-MS). Peak intensity is shown with A.U.= arbitrary units. *n* = 5 independent experiments. **d**, qRT-PCR showing expression levels of the transcriptional marker of *Fh1* loss, *Hmox1,* in *iFh1*^{CL33} cells. *n* = 5 independent experiments. Bar graphs show the fold change expression, for which the expression in control samples was set to 1. Indicated p-

values are relative to the corresponding vehicle (EtOH)-treated time point. **e**, qRT-PCR showing expression levels of *Ifnb1* and Interferon-stimulated genes (ISGs) (*Ifi202b*, *Ifitm10*, *Areg* and *Ccl20*) in $iFh1^{CL33}$ cells. n = 5 independent experiments. Bar graphs show the fold change expression, for which the expression in control samples was set to 1. Indicated p-values are relative to the corresponding EtOH-treated time point. **f**,**g**, Percentage of $iFh1^{CL33}$ cells showing cytosolic DNA *foci* (**f**), and number of cytosolic DNA *foci* per cell (**g**). n = 3 independent experiments. **h**-**j**, Classification of mitochondrial morphology (**h**), quantification of mitochondria number (**i**), and area (**j**), in $iFh1^{CL33}$ cells. n = 3 independent experiments. Indicated p-values are relative to the corresponding vehicle (EtOH)-treated morphology category. **k**-**m**, Quantification of mtDNA copy number by digital droplet PCR (ddPCR) using either a mtCo3 (**k**), ND1 (**l**) or D-loop (**m**) probe, from isolated cytosolic fractions of $iFh1^{CL33}$ cells. n = 3 independent experiments. Data are mean ± s.e.m. **a**,**c**-**e**, Students t-test corrected for multiple comparison with the Holm-Sidak method, **f**,**g**,**i**-**m**, one-way ANOVA with Tukey's multiple comparison test, **h**, two-way ANOVA with Tukey's multiple comparison test.

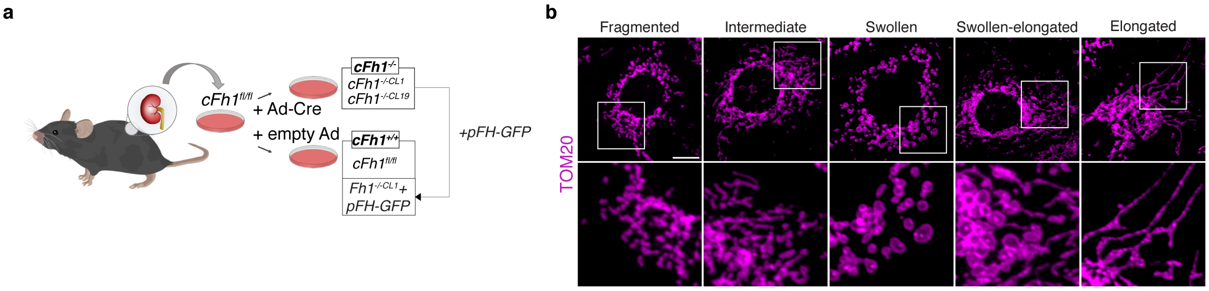

Supplementary Figure 2

a, Schematic diagram for the generation of two epithelial kidney cell lines with chronic *Fh1* deletion (*cFh1*$^{-/-CL1}$ and *cFh1*$^{-/-CL19}$) from cFh1$^{fl/fl}$. Ad-Cre = adenovirus-Cre. pFH = human FH-expressing exogenous plasmid. **b**, Representative confocal images of mitochondrial morphology (TOM20) in *cFh1*$^{-/-\text{CL19}}$ cells with indicated mitochondrial morphology phenotypes. Scale bar: 10 μm.

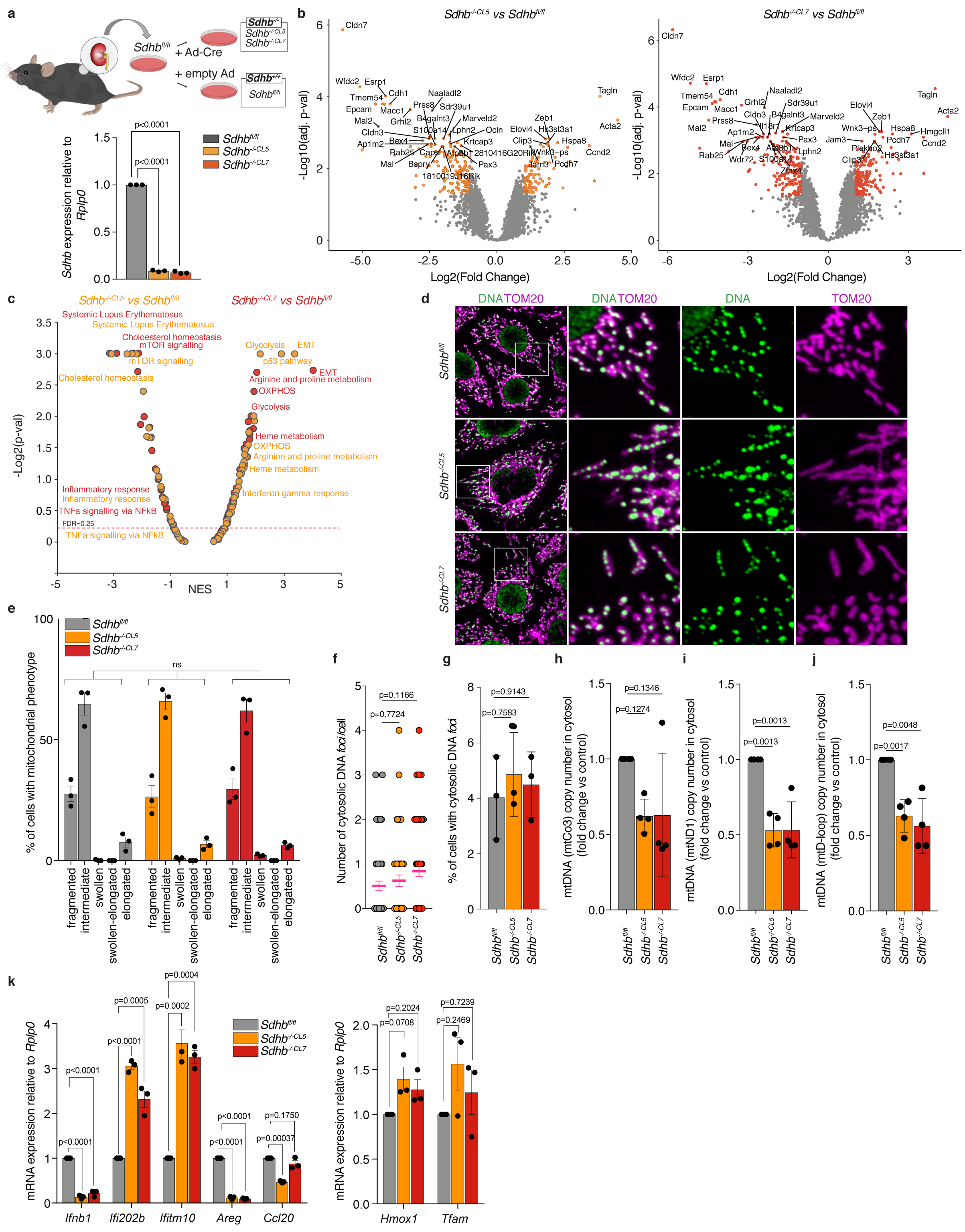

Supplementary Figure 3

a, Top: Schematic diagram for the generation of two epithelial kidney cell lines with chronic *Sdhb* deletion (*cSdhb*$^{-/-CL5}$ and *cSdhb*$^{-/-CL7}$) derived from the *cSdhb*$^{fl/fl}$ line[34]. Bottom: qRT-PCR showing expression levels of *Sdhb* in *cSdhb*$^{-/-CL5}$ and *cSdhb*$^{-/-CL7}$ compared to *cSdhb*$^{fl/fl}$ measured by qRT-PCR; Ad-Cre = adenovirus-Cre. $n = 3$ independent experiments. **b**, Volcano plots showing the disregulated genes in *cSdhb*$^{-/-CL5}$ (left) and *cSdhb*$^{-/-CL7}$ *vs Sdhb*$^{fl/fl}$ kidney cell

lines. **c**, Volcano plot for the Gene Set Enrichment Analysis highlighting the differentially regulated pathways in *cSdhb*$^{-/-CL5}$ (orange) and *cSdhb*$^{-/-CL7}$ (red) *vs* *Sdhb*$^{fl/fl}$ kidney cell lines. NES=Normalised Enrichment Score. **d**, Representative confocal images of mitochondrial morphology (TOM20) and DNA *foci* (DNA) in *cSdhb* cells. Scale bar: 10 µm. **e**, Quantification of mitochondrial morphology in *cSdhb* cell lines. $n = 3$ independent experiments. **f**,**g**, Number of cytosolic DNA *foci* per cell (**f**), and percentage of cells with cytosolic DNA (**g**), in *cSdhb* cells from **d**. $n = 3$ independent experiments. **h**-**j**, Quantification of mtDNA copy number by ddPCR using either a mtCo3 (**h**), mtND1 (**i**), or D-loop (**j**) probe, from isolated cytosolic fractions of *cSdhb* cell lines. $n = 3$ independent experiments. **k**, Expression of a panel of ISGs (left) and negative controls *Hmox1* and *Tfam* (right), in c*Sdhb* cells measured by qRT-PCR. $n = 3$ independent experiments. Bar graphs show the fold change expression, for which the expression in control samples was set to 1. Data are mean ± s.e.m. **a**,**f**-**k**, one-way ANOVA with Tukey's multiple comparison test, **e**, two-way ANOVA with Tukey's multiple comparison test.

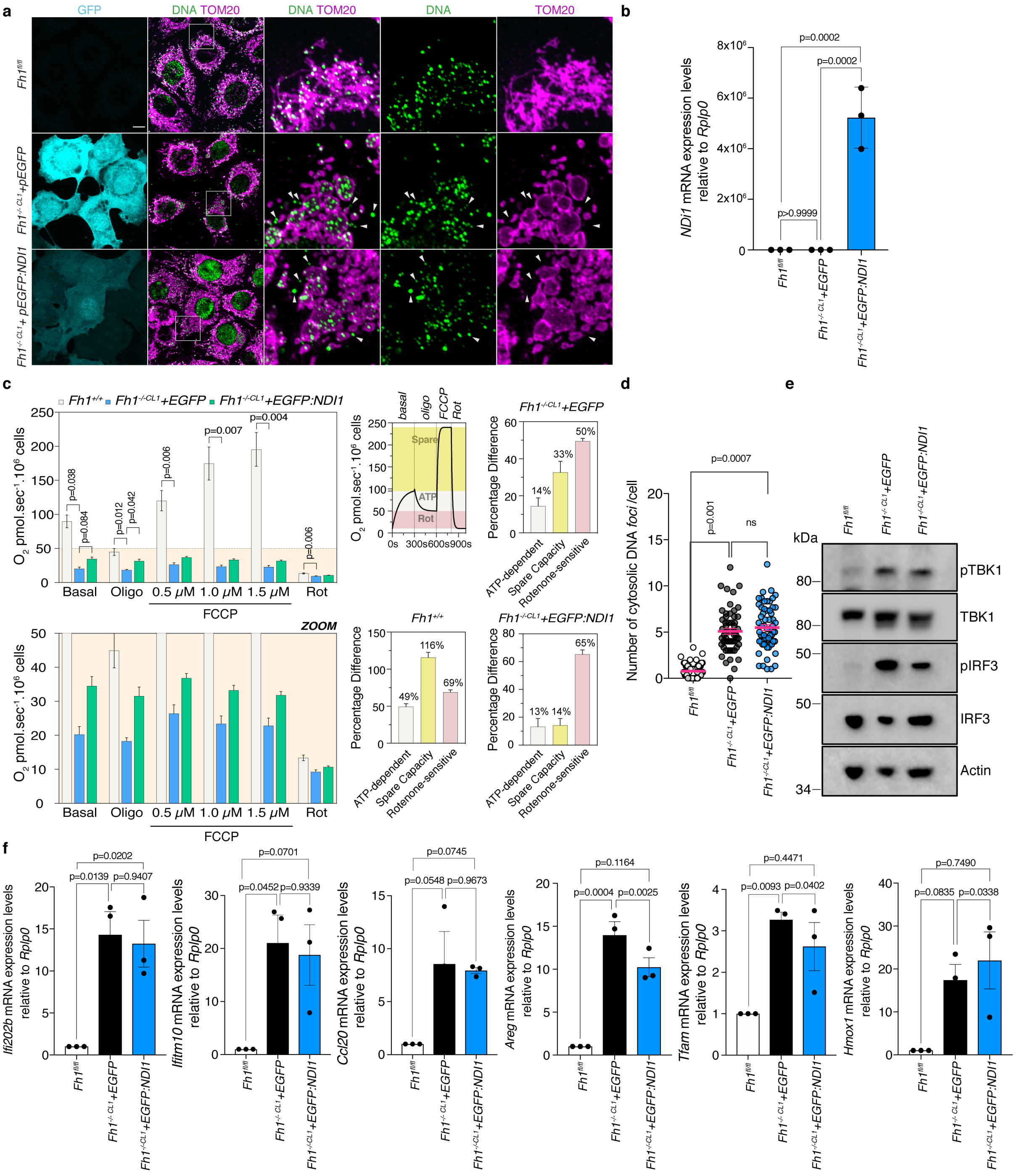

Supplementary Figure 4

a, Representative confocal images of *cFh1*$^{fl/fl}$ and *cFh1*$^{-/-CL1}$ cells stably expressing pEGFP (*cFh1*$^{-/-CL1}$*+EGFP*) or pEGFP:NdI1 (*cFh1*$^{-/-CL1}$*+EGFP:NDI1*). Mitochondria and DNA were labelled using anti-TOM20 and anti-DNA antibodies, respectively. White arrows indicate cytosolic DNA *foci*. Scale bar: 10 µm. **b**, mRNA expression of *NDI1* in *cFh1* cells measured by qRT-PCR. *n* = 3 independent experiments. Bar graphs show the fold change expression, for which the expression in control samples was set to 1. **c**, Quantification of oxygen consumption in *cFh1* cells with Oroboros (left panels); panels on the right represent the percentage of ATP

dependent, spare capacity and rotenone sensitive oxygen consumption. *n* = 4 independent experiments. **d**, Number of cytosolic DNA *foci* in *cFh1* cells from **a**. *n* = 3 independent experiments. **e**, Immunoblots of specified proteins in *cFh1* cells. **f**, Expression of the ISGs *Ifi202b*, *Ifitm10*, *Ccl20* and *Areg*; and negative and positive controls *Tfam* and *Hmox1*, respectively, in *cFh1* cells. *n* = 3 independent experiments. Bar graphs show the fold change expression, for which the expression in control samples was set to 1. Data are mean ± s.e.m. **b**,**d**,**f**, one-way ANOVA with Tukey's multiple comparison test.

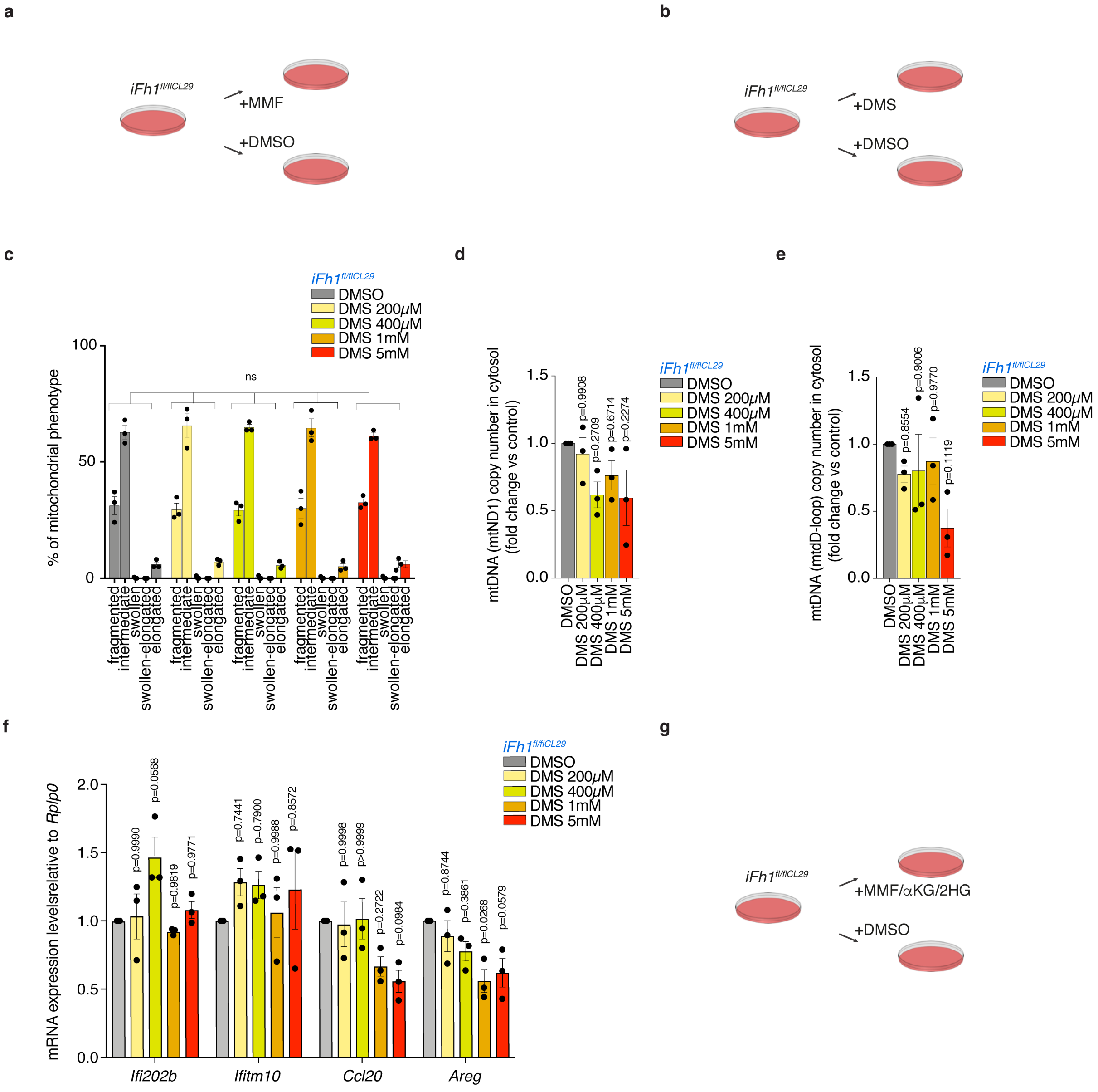

Supplementary Figure 5

a, Schematic diagram showing cell permeable form of fumarate, monomethylfumarate (MMF), treatment of inducible *iFh1* epithelial kidney cell lines clones 29 (*iFh1*$^{fl/flCL29}$). **b**, schematic diagram showing cell permeable form of succinate, dimethylsuccinate (DMS), treatment of *iFh1*$^{fl/flCL29}$. **c**, Classification of mitochondrial morphology in *iFh1*$^{fl/flCL29}$ cells treated with vehicle (DMSO) or DMS at the indicated concentration for 8 days. *n* = 3 independent experiments. **d**,**e**, Quantification of mtDNA copy number by ddPCR using either a ND1 (**d**) or D-loop (**e**) probe, from isolated cytosolic fractions of *iFh1*$^{fl/flCL29}$ cells treated with DMSO or DMS. *n* = 3 independent experiments. **f**, Expression of the ISGs *Ifi202b*, *Ifitm10*, *Ccl20* and *Areg* in *iFh1*$^{fl/flCL29}$ cells treated with DMSO or DMS. *n* = 3 independent experiments. **g**, Schematic diagram showing MMF, αKG or 2-HG treatment of *iFh1*$^{fl/flCL29}$ cells. Data are mean

± s.e.m. **c**, two-way ANOVA with Tukey's multiple comparison test, **d**-**f**, one way ANOVA with Tukey's multiple comparison test, bar graphs show the fold change expression, for which the expression in control samples was set to 1. p-values indicated above each bar are relative to the DMSO control.

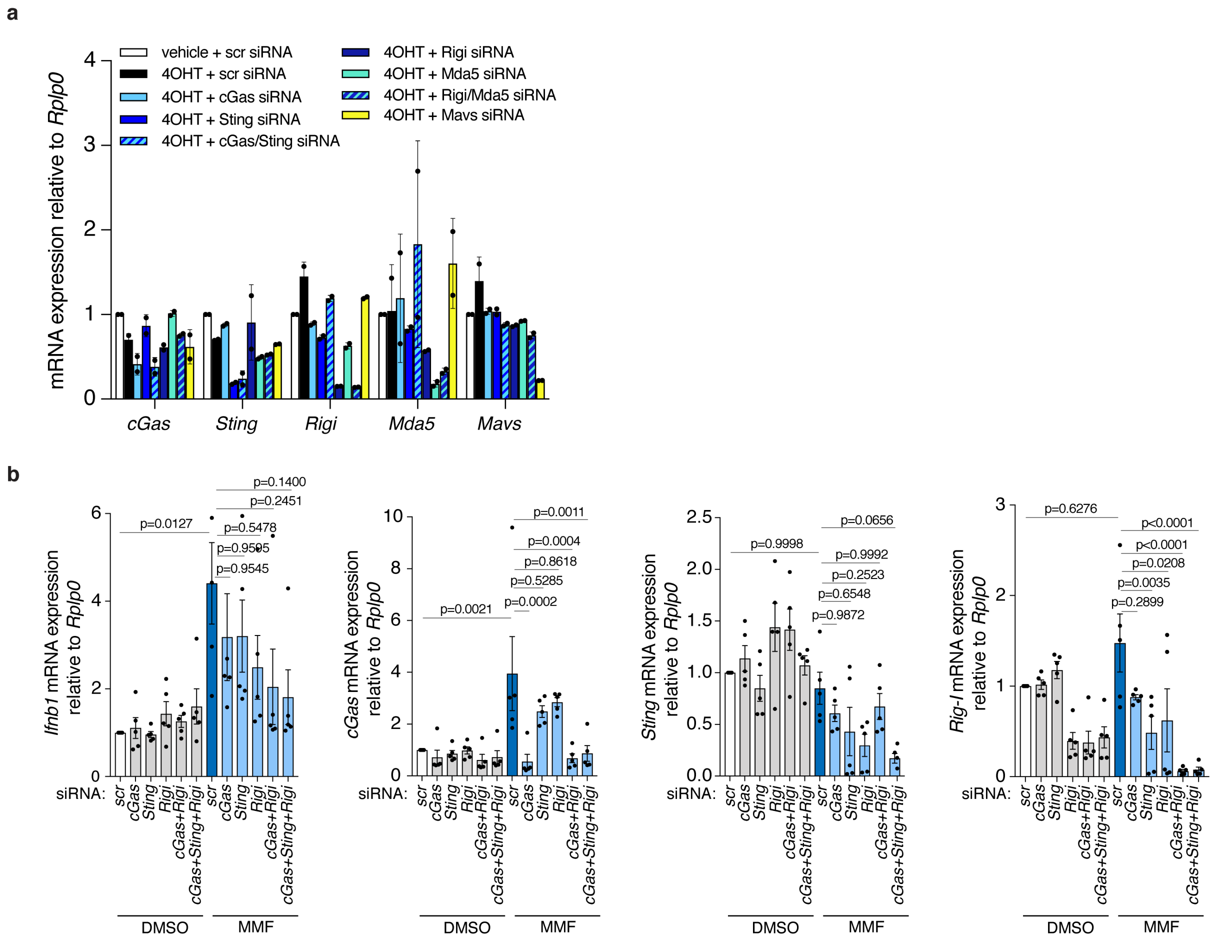

Supplementary Figure 6

a, mRNA expression of siRNA-targeted genes in *iFh1*^{cl29} cells at 15 days post induction transfected with indicated siRNA measured by qRT-PCR. *n* = 2 independent experiments. Bar graphs show the fold change expression, for which the expression in control samples was set to 1. **b**, mRNA expression of *Ifnb1* and siRNA-targeted genes *cGas*, *Sting*, and *Rig-I* in *iFh1*^{CL29} cells, treated with either DMSO or MMF for 8 days, and with combinations of siRNAs for *cGas*, *Sting* and *Rig-I*; measured by qRT-PCR. *n* = 4 independent experiments. Bar graphs show the fold change expression, for which the expression in control samples was set to 1. Data are mean ± s.e.m. **b**, one-way ANOVA with Tukey's multiple comparison test.

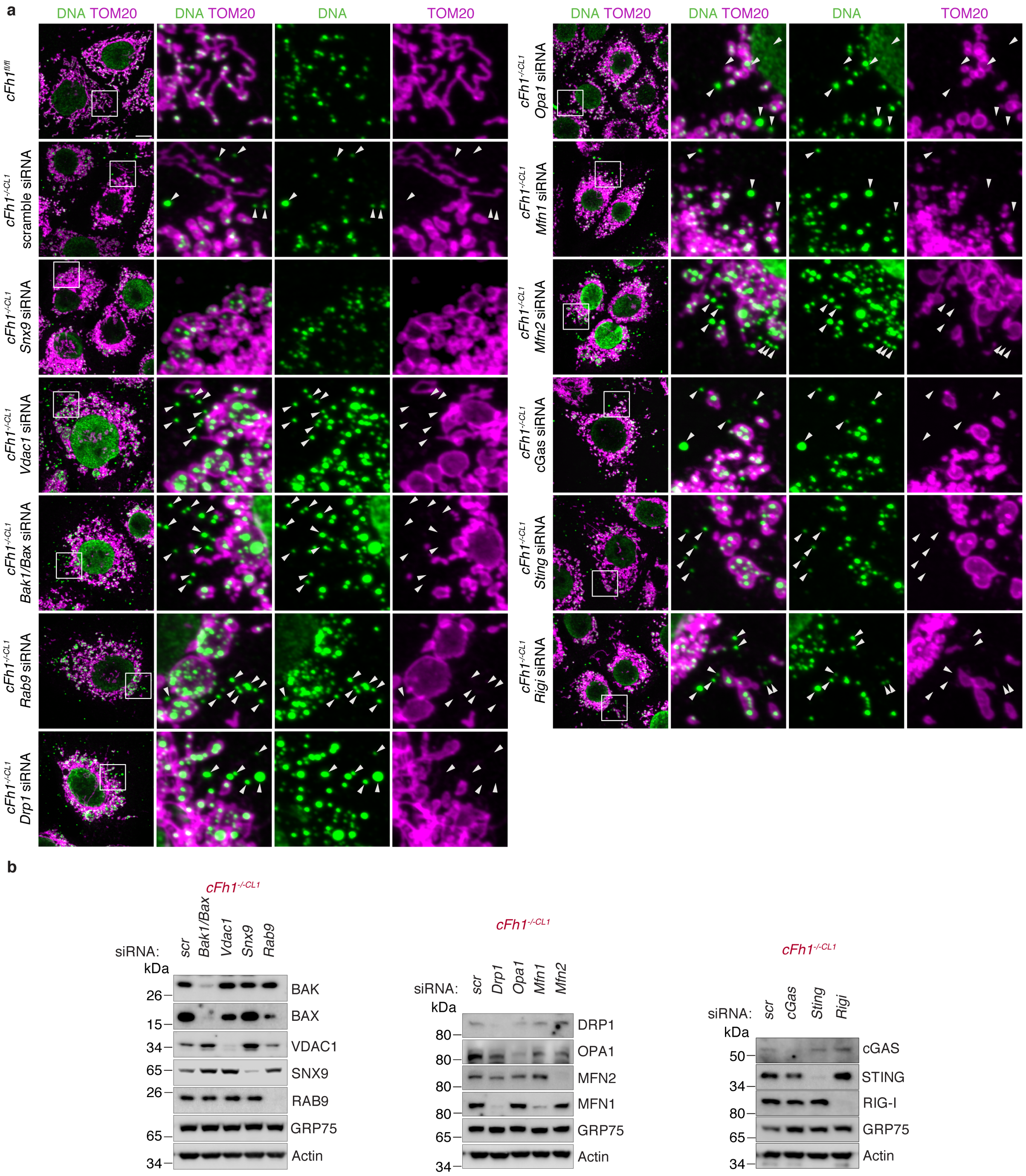

Supplementary Figure 7

a, Representative confocal images of mitochondrial morphology (TOM20) and DNA *foci* (DNA) in *cFh1*$^{-/-CL1}$ cells untreated or treated with indicated siRNA. White arrows indicate cytosolic DNA *foci*. scale bar: 10 µm. **b**, Immunoblots of specified proteins in *cFh1*$^{-/-CL1}$ cells treated with indicated siRNA.

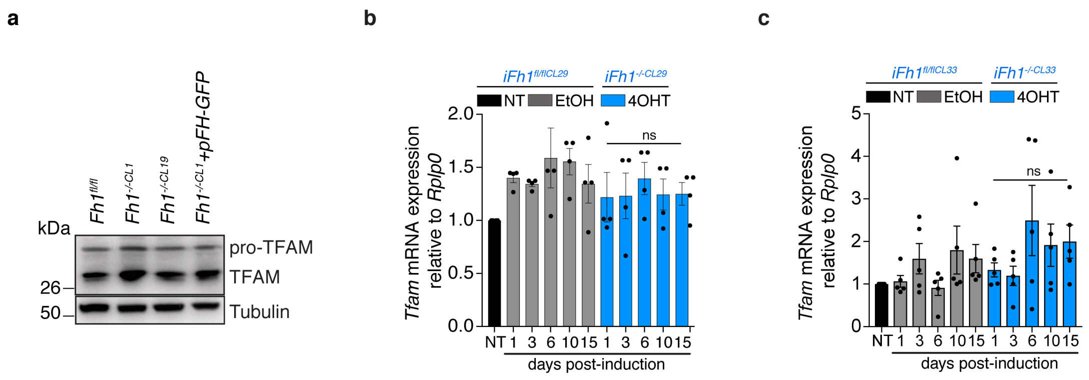

Supplementary Figure 8

a, Immunoblots of pro-TFAM and TFAM in *cFh1* cells. **b**,**c**, *Tfam* expression in untreated (NT), vehicle (EtOH)- or 4-hydroxytamoxifen (4-OHT)-treated *iFh1*$^{fl/flCL29}$ (left) and *iFh1*$^{fl/flCL33}$ (right) cells, measured by qRT-PCR. n = 5 independent experiments. Bar graphs show the fold change expression, for which the expression in control samples was set to 1. Data are mean ± s.e.m. **b**,**c**, Students t-test corrected for multiple comparison with the Holm-Sidak method.

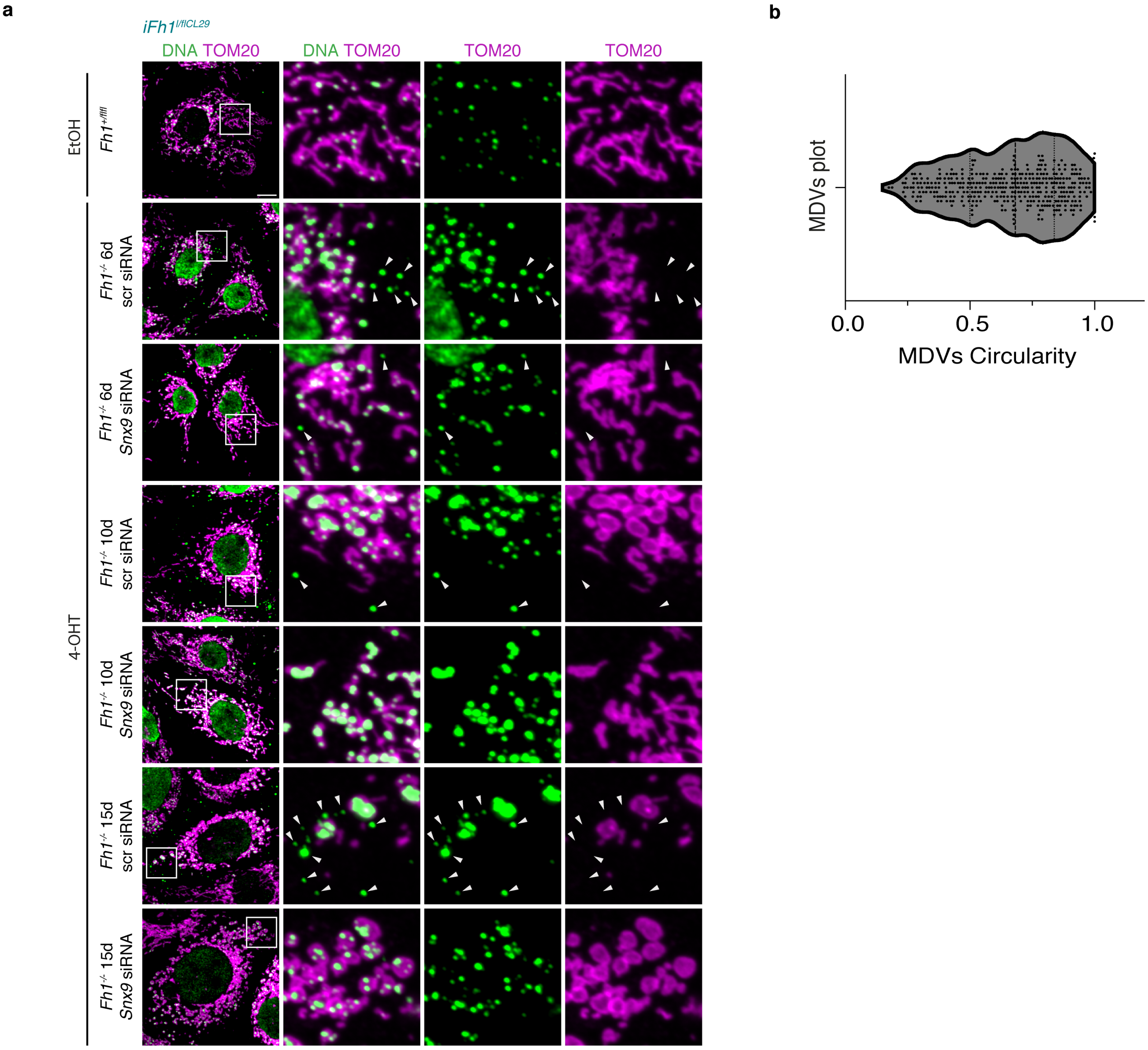

Supplementary Figure 9

a, Representative confocal images of mitochondrial morphology (TOM20) and DNA *foci* (DNA) in vehicle (EtOH)- or 4-hydroxytamoxifen (4-OHT)-treated *iFh1*$^{fl/flCL29}$ cells transfected with scramble (scr) or *Snx9* siRNA for the indicated period of time. White arrows indicate cytosolic DNA *foci*. Scale bar: 10 µm. **b**, Quantification of the circularity of TOM20^{-} PDH^{+}DNA^{+} MDVs obtained from lattice super resolution SIM image of MMF-treated *cFh1*$^{fl/fl}$ cells. n = 3 independent experiments.

Uncropped Immunoblots: Main Figures

Figure 2a

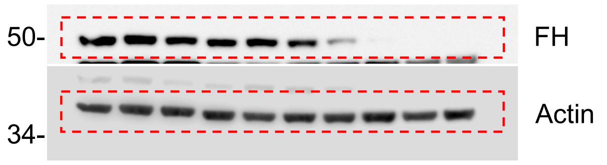

Figure 2c

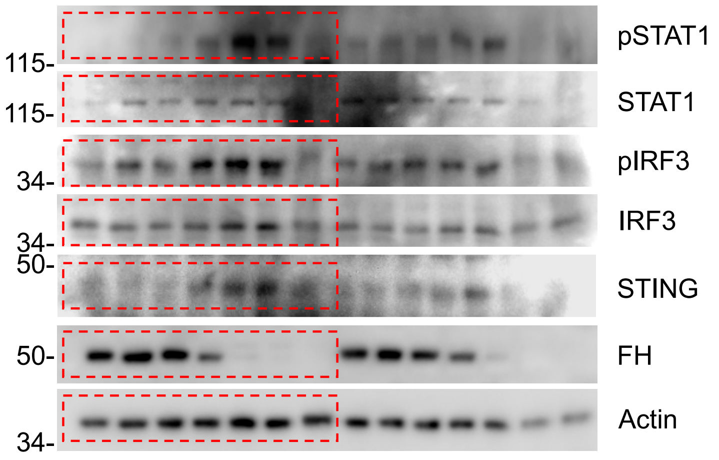

Figure 3e

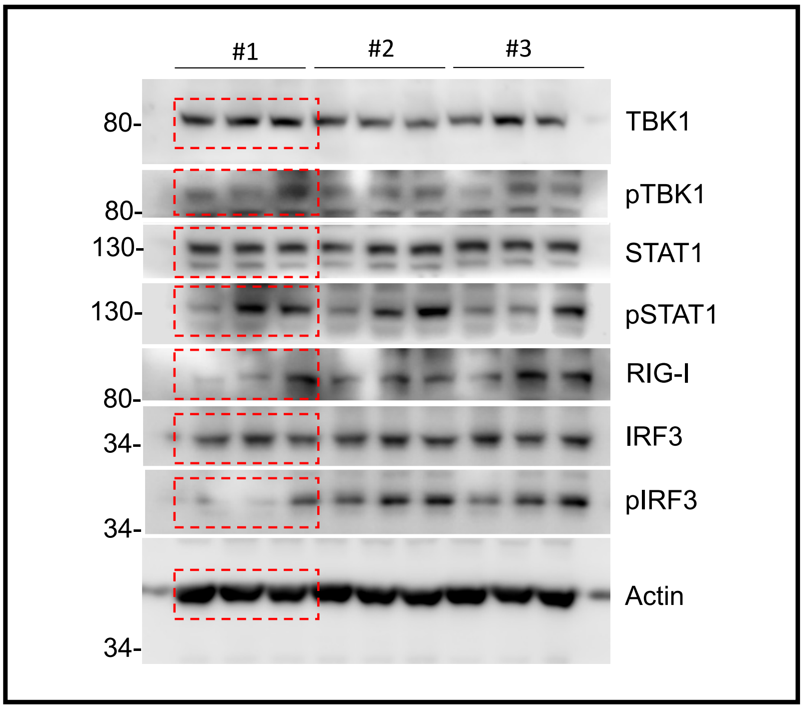

Figure 3g

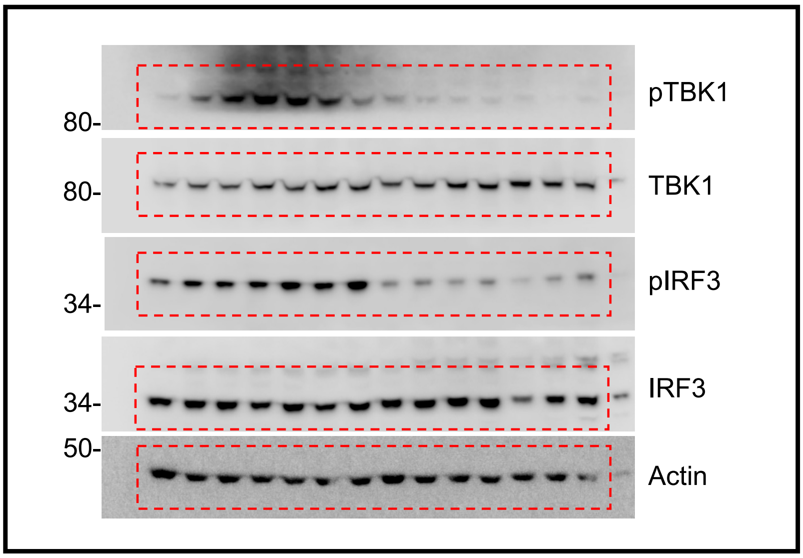

Figure 3i

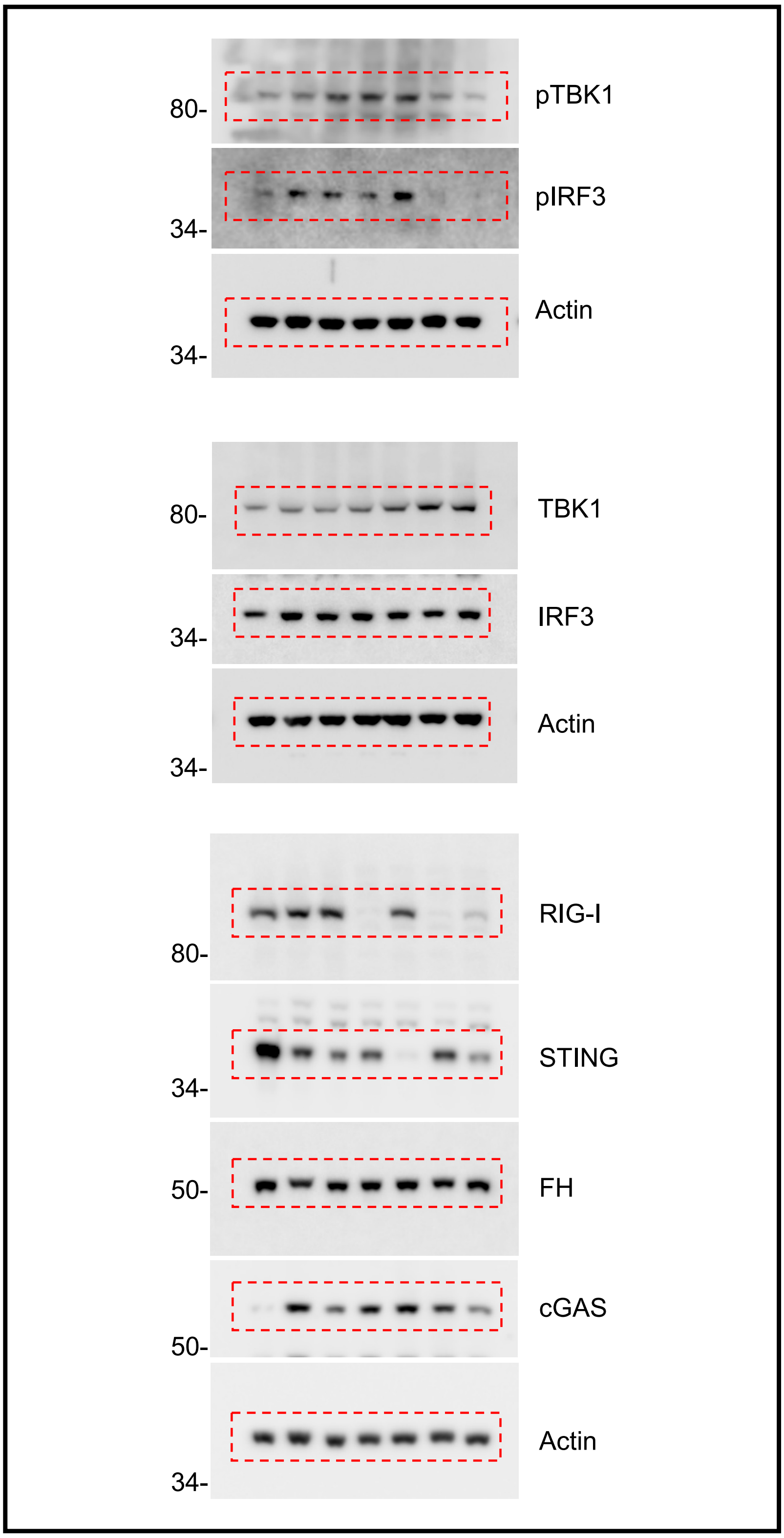
80-
pTBK1
pIRF3
34-
Actin
34-
80-
TBK1
IRF3
34-
Actin
34-
RIG-I
80-
STING
34-
50-
FH
cGAS
50-
Actin
34-

Figure 4J

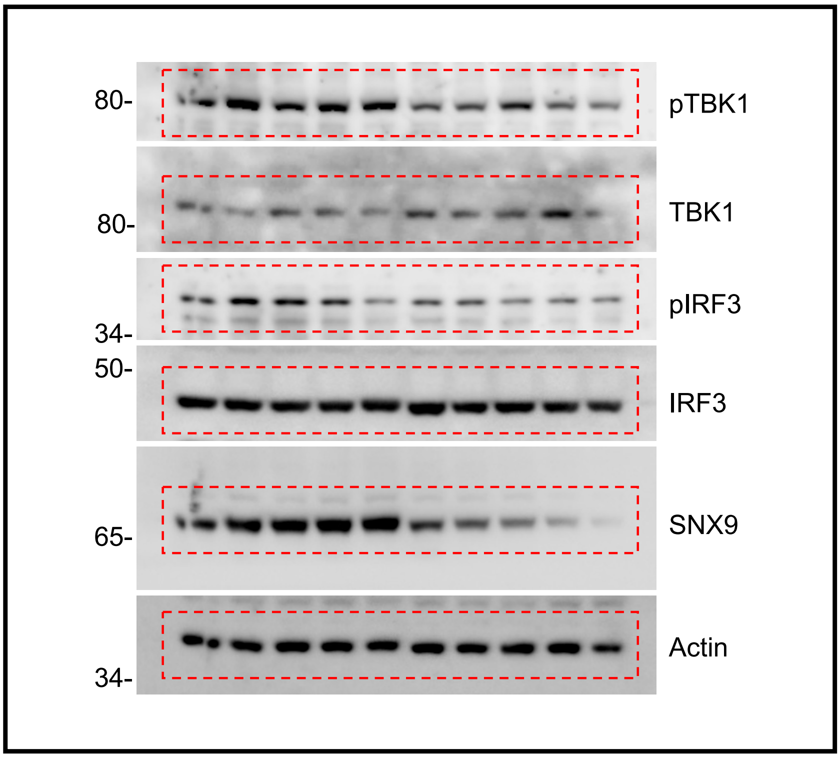

Uncropped Immunoblots: Extended Data Figures

Figure ED1a

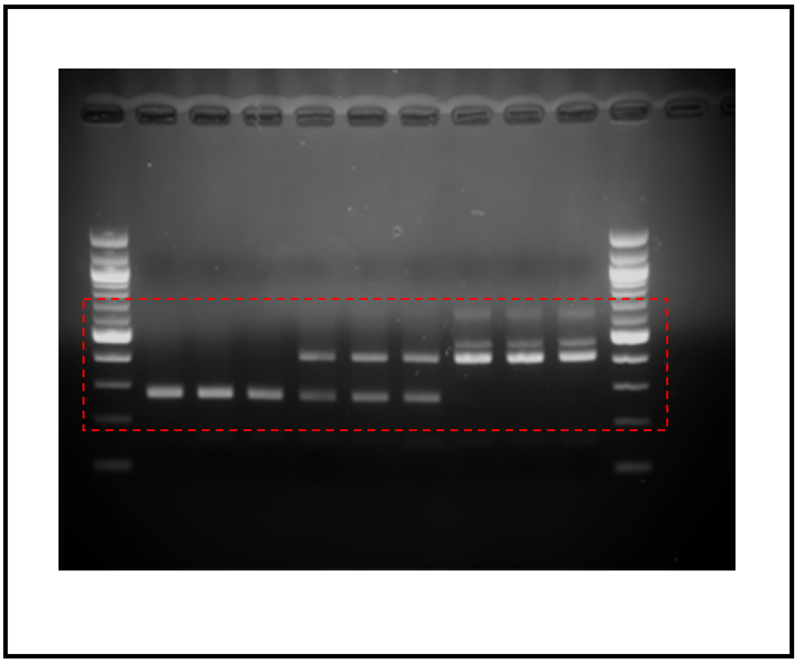

Figure ED1b

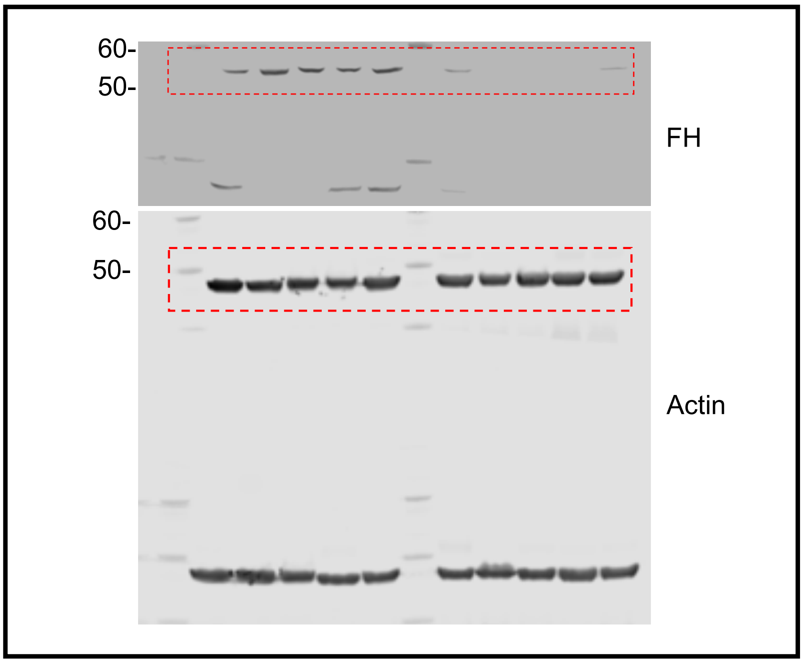

Figure ED3k

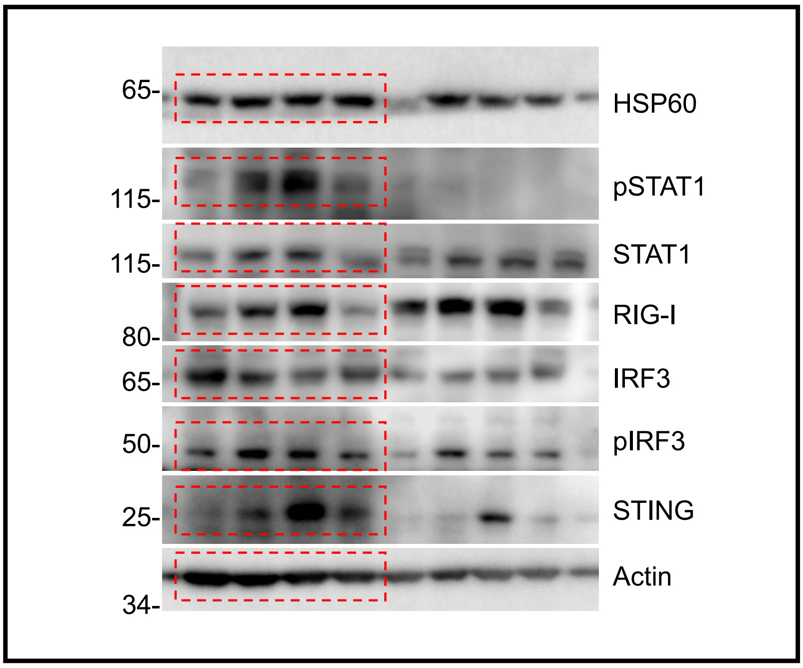

Figure ED6h

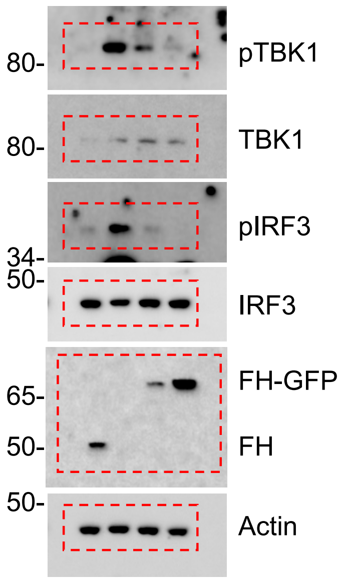

Figure ED7e

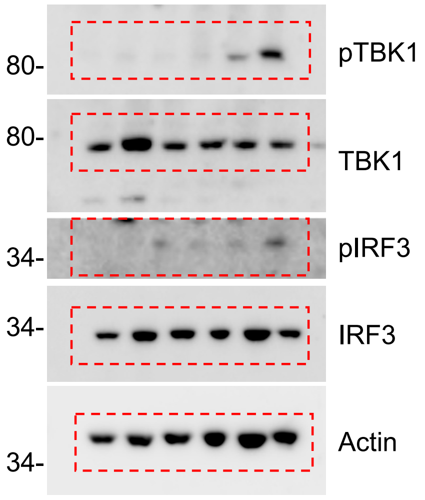

Figure ED7f

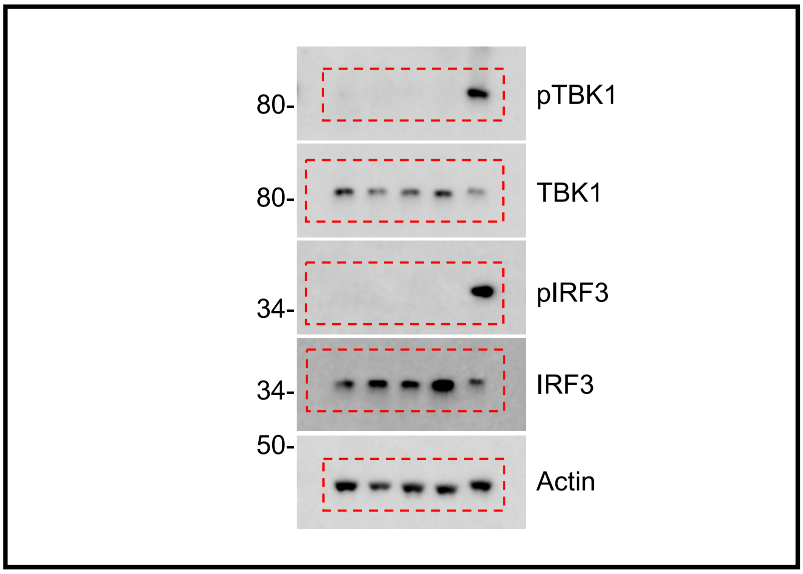

Figure ED8i

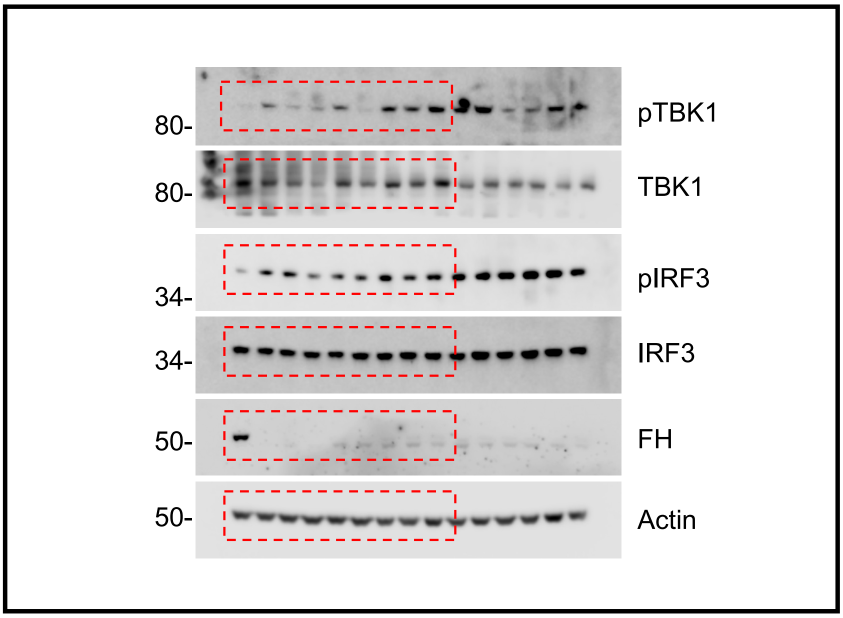

Figure ED9f

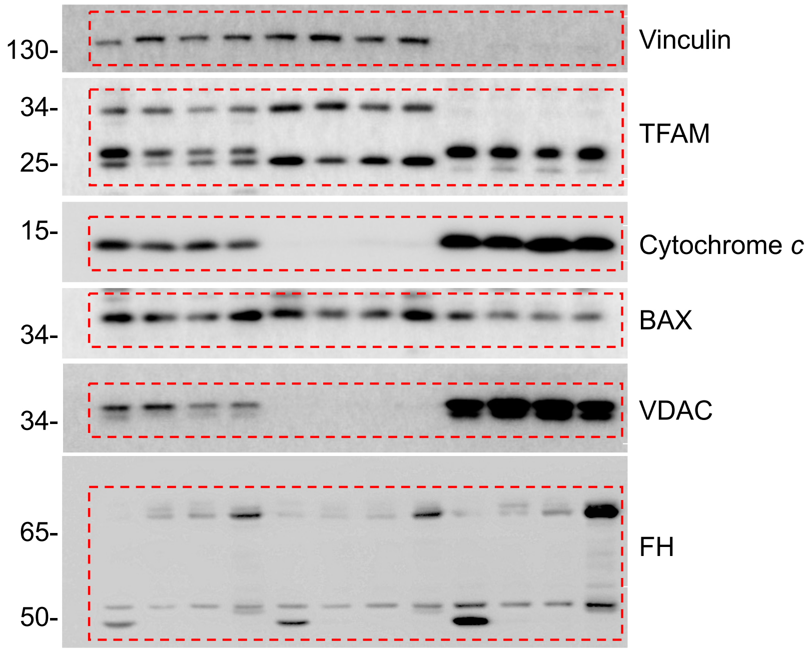

Figure ED10d

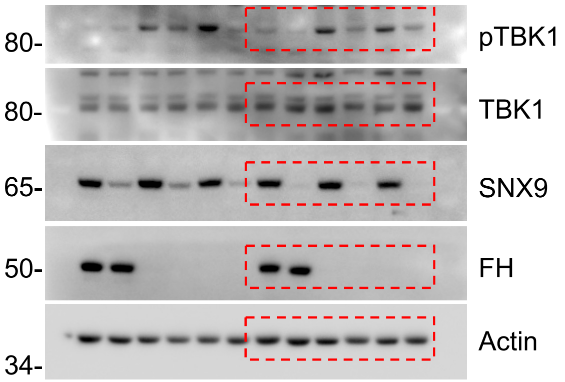

Figure ED10i

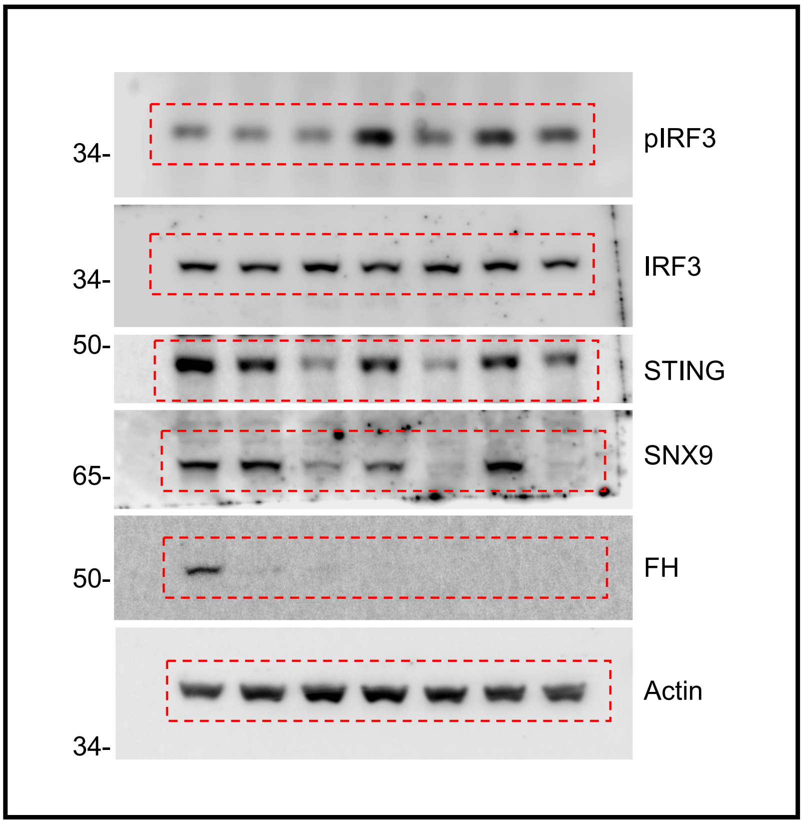

Uncropped Immunoblots: Supplementary Figures

Supplementary Figure 4e

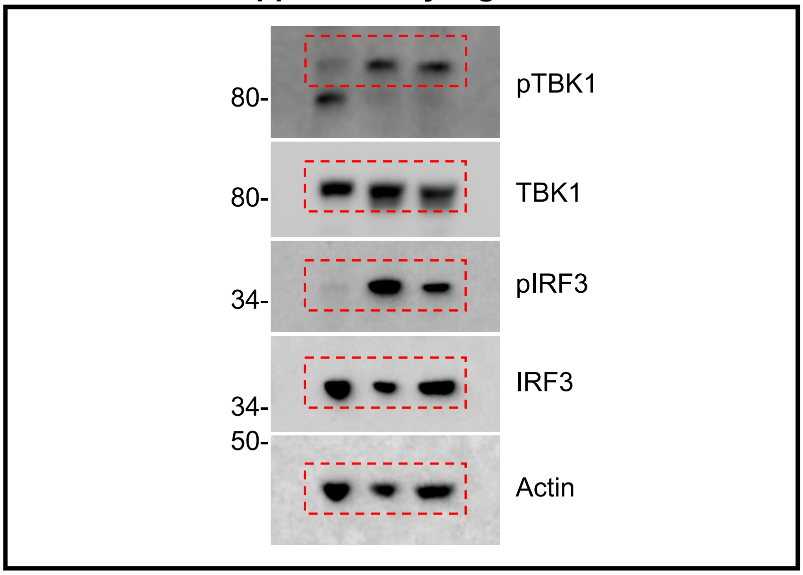

Supplementary Figure7b

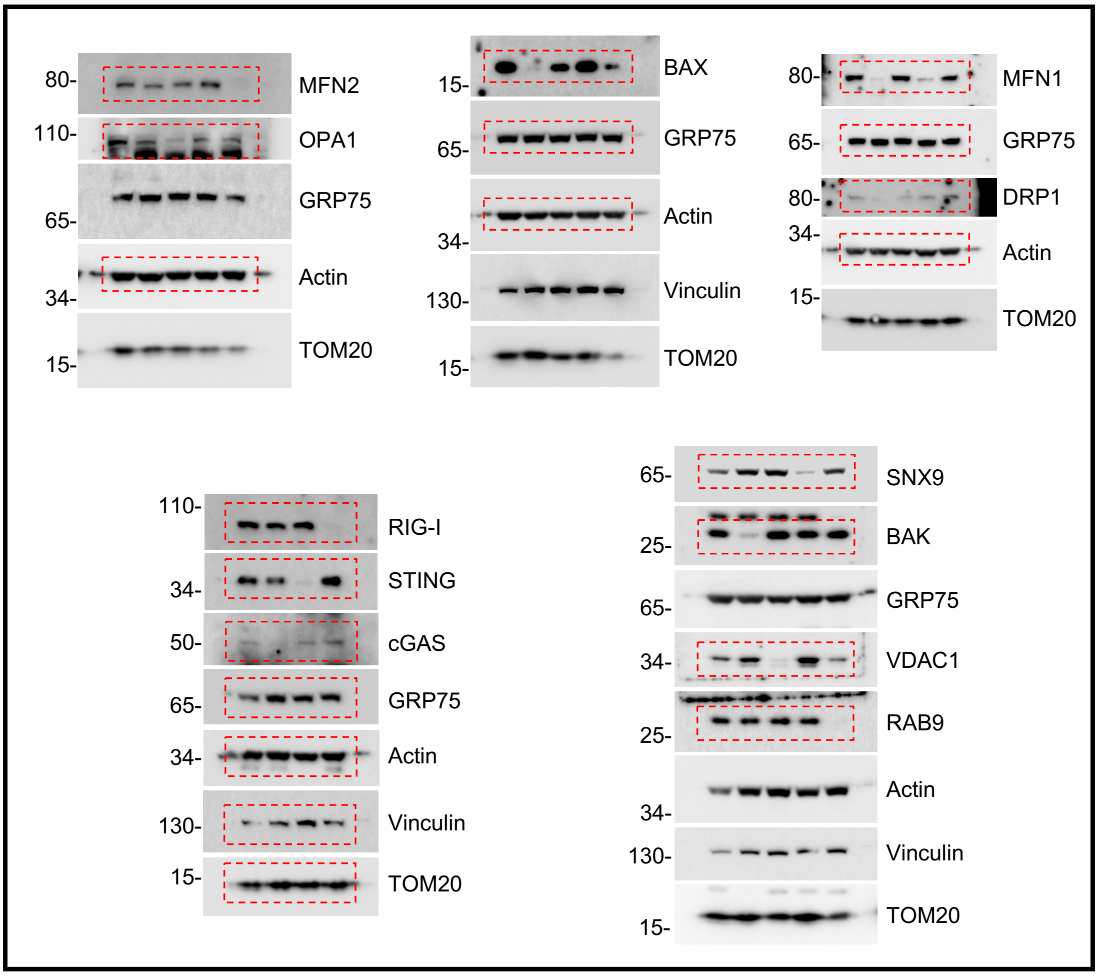

Supplementary Figure 8a

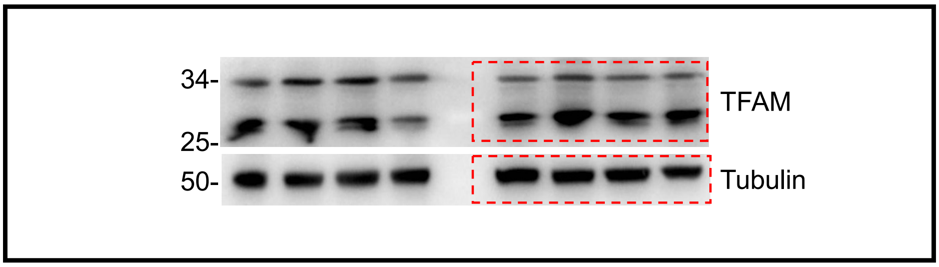

Supplementary Figure 10

Uncropped gels and immunoblots from figures.

Supplementary Table 1: RNASeq analysis of the *Fh1*$^{-/-}$ *vs Fh1*$^{+/+}$ mouse kidney at Days 5 and 10 upon tamoxifen treatment. See Excel sheet 1.

Supplementary Table 2: Biological processes analysis of the *Fh1*$^{-/-}$ *vs Fh1*$^{+/+}$ mouse kidney at Day 5 upon tamoxifen treatment. See Excel sheet 2.

Supplementary Table 3: KEGG pathway analysis of the *Fh1*$^{-/-}$ *vs Fh1*$^{+/+}$ mouse kidney at Day 5 upon tamoxifen treatment. See Excel sheet 3.

Supplementary Table 4: Mouse primers list.

Target	forward	reverse
Mm Areg	TGGCAGTGAACTCTCCACAG	TTCTTGGGCTTAATCACCTGTT
Mm Ccl17	AGTGGAGTGTTCCAGGGATG	TGGCCTTCTTCACATGTTTG
Mm Ccl2	AAGAGGATCACCAGCAGCAG	TCTGGACCCATTCCTTCTTG
Mm Ccl20	TTTTTGGGATGGAATTGGAC	AGGTCTGTGCAGTGATGTGC
Mm cGas	TCAGCTACCAAGATGCTGTCA	GGCTTCCTGGTTTTTCCTTC
Mm Cxcl10	CCAAGTGCTGCCGTCATTTTC	GGCTCGCAGGGATGATTTCAA
Mm Fh1 ex2-3	AACGTATGCCAATCCCAGTC	CATCTGCGGCCTTCATTATT
Mm Hmgb1	TGACAAGCAGCCCTATGAGA	CTTTTTCGCTGCATCAGGTT
Mm Hmox1	GGTCAGGTGTCCAGAGAAGG	GCTTGTTGCGCTCTATCTCC
Mm Ifi202b	GAAAGGCTGGTTGATGGAGA	CCACCACTTTCATTGCTCCT
Mm Ifitm10	CGAGGTCTACCCGGACACTA	GTAGGCCAGAGCAATGAAGC
Mm Ifnb	CCCTATGGAGATGACGGAGA	CCCAGTGCTGGAGAAATTGT
Mm Il6	CCGGAGAGGAGACTTCACAG	TCCACGATTTCCCAGAGAAC
Mm Mx1	GGTCCAAACTGCCTTCGTAA	TTCAGCTTCCTTTTCTTGGTTT
Mm Mx1	GGTCCAAACTGCCTTCGTAA	TTCAGCTTCCTTTTCTTGGTTT
Mm Nqo1	AGGCTGGTTTGAGAGAGTGC	CCCCAGTGGTGATAGAAAGC
Mm Rplp0	GATTCGGGATATGCTGTTGG	TCGGGTCCTAGACCAGTGTT
Mm Snx9	GCACAGCTCAAACCAACTCA	AGGTTTGGGAGCATTCCAG
Mm Snx9	GCACAGCTCAAACCAACTCA	AGGTTTGGGAGCATTCCAG
Mm Tfam	AAGGATGATTCGGCTCAGG	GGCTTTGAGCACTAACTGG

Supplementary table 5: Human primers list.

target	forward	reverse
AREG	CGGGAGCCGACTATGACTAC	GGGGGCTTAACTACCTGTTCA
CCL2	GCCTCCAGCATGAAAGTCTC	GTGACTGGGGCATTGATTG
CCL20	TTTATTGTGGGCTTCACACG	GATTTGCGCACACAGACAAC
CSF3	GTGCTGCTCGGACACTCTCT	TAGAGGAAAAGGCCGCTATG
FH	TGCAATAATGAAGGCAGCAG	TGATCCAGTCTGCCATACCA
IFI16	TCTTCACAAACAAGCTTCAGGA	CCTGAATTTCGTAGATTGTGGTC
IFITM10	CTTCATCGCCTTGGCCTACT	GGCAGAACTGGTGATGTTGA
