# Supplementary material for: Fumarate induces vesicular release of mtDNA to drive innate immunity
Source: Nature. 2023 Mar 8;615(7952):499–506. doi: 10.1038/s41586-023-05770-w (PMC10017517; doi:10.1038/s41586-023-05770-w)
Supplement: Supplementary file 2 — Reporting Summary [file 41586_2023_5770_MOESM2_ESM.pdf]

nature portfolio

Corresponding author(s): Christian Frezza

Last updated by author(s): Dec 8, 2022

# Reporting Summary

Nature Portfolio wishes to improve the reproducibility of the work that we publish. This form provides structure for consistency and transparency in reporting. For further information on Nature Portfolio policies, see our Editorial Policies and the Editorial Policy Checklist.

## Statistics

For all statistical analyses, confirm that the following items are present in the figure legend, table legend, main text, or Methods section.

| n/a | Confirmed | |
|---|---|---|
| ☐ | ☒ | The exact sample size (*n*) for each experimental group/condition, given as a discrete number and unit of measurement |
| ☐ | ☒ | A statement on whether measurements were taken from distinct samples or whether the same sample was measured repeatedly |
| ☐ | ☒ | The statistical test(s) used AND whether they are one- or two-sided<br>*Only common tests should be described solely by name; describe more complex techniques in the Methods section.* |
| ☐ | ☐ | A description of all covariates tested |
| ☐ | ☐ | A description of any assumptions or corrections, such as tests of normality and adjustment for multiple comparisons |
| ☐ | ☐ | A full description of the statistical parameters including central tendency (e.g. means) or other basic estimates (e.g. regression coefficient) AND variation (e.g. standard deviation) or associated estimates of uncertainty (e.g. confidence intervals) |
| ☐ | ☒ | For null hypothesis testing, the test statistic (e.g. *F*, *t*, *r*) with confidence intervals, effect sizes, degrees of freedom and *P* value noted<br>*Give P values as exact values whenever suitable.* |
| ☐ | ☐ | For Bayesian analysis, information on the choice of priors and Markov chain Monte Carlo settings |
| ☐ | ☐ | For hierarchical and complex designs, identification of the appropriate level for tests and full reporting of outcomes |
| ☐ | ☐ | Estimates of effect sizes (e.g. Cohen's *d*, Pearson's *r*), indicating how they were calculated |

*Our web collection on statistics for biologists contains articles on many of the points above.*

## Software and code

Policy information about availability of computer code

Data collection

All softwares used for data collection are publicly or commercially available:
H&E stained tissue slides were generated using an Axioscan Z1 and ZEN Blue (v3.1) software. Immunofluorescence images were acquired using a Nikon Eclipse TiE inverted microscope with Andor Dragonfly 500 spinning disk system, equipped with a Zyla 4.2 PLUS sCMOS camera (Andor) coupled with Fusion software and using a 100X objective lens (NA1.4). Super-resolution images were acquired with an N-Structured illuminated Microscopy (N-SIM) microscope using a SR Apo TIRF 100× 1.49 N.A. oil objective and a DU897 Ixon camera (Andor). Raw images were computationally reconstructed using the reconstruction slice system from NIS-Elements software (Nikon). TFAM+-MDVs-related super-resolution images (Extended Data Fig. 12a) were acquired with a Zeiss LSM 880 microscope with an Airyscan detector (Carl Zeiss Microscopy, Jena, Germany), using a Zeiss 63x oil lens, numerical aperture 1.4, as the primary objective. Four colour excitation was performed with a Blue diode laser for 405 nm, Argon laser for 488 nm, He 543 laser for 561 nm and He 633 laser for 647 nm.. The Airyscan detector was used in SR mode utilising all 32 pinholes, thus increasing resolution to ~140 nm in x, y and z to capture the image. The image was reconstructed by pixel reassignment and deconvolution on Zen Black platform (Carl Zeiss Microscopy, Jena, Germany). The final image was produced on ImageJ by background subtraction (rolling ball 50) and one run of the smooth filter. MDVs super-resolution mages were acquired with a Zeiss Elyra7 equipped with Lattice SIM2 (Carl Zeiss Microscopy, Jena, Germany) with a 15 phased imaging protocol for all channels (Fig. 4h). A Zeiss 63x OIL: Plan-Apo 63x/1.4 Oil Corr WD: 0.35, was used as the primary objective to obtain representative images of individual cells. The images used for quantification of MDVs dimensions and number were acquired on a 40x OIL: Plan-APO 40x/1.4 Oil DIC (UV) VIS-IR objective using the SIM Apotome mode to maximize field of view and include more than one cell at a higher resolution.Electron microscopy images were acquired using a Tecnai G2 (FEI) transmission electron microscope operating at 100 kV equipped with a Veleta (Olympus Soft Imaging System) digital camera. RNA sequencing was performed using an Illumina HiSeq4000 sequencer. In vivo samples LC-MS was performed using a Dionex U3000 UHPLC system coupled to a Q Exactive mass spectrometer (Thermo Fisher Scientific) with a Sequant ZIC-pHILIC column (Merck Millipore). For in vitro experiments, LC-MS was performed on a QExactive Orbitrap mass spectrometer coupled to a Dionex UltiMate 3000 Rapid Separation LC system (Thermo Fisher Scientific), fitted with either a SeQuant Zic-HILIC column or a SeQuant Zic-pHILIC. Quantitative-RT

PCR was performed using an Applied Biosystems StepOne Plus or QuantSudio5 real-time PCR system. Genomic DNA amplification PCR was performed on Applied Biosystems Verity Thermo Cycler. Digital droplet PCR was performed using MODEL ASK VP (Biorad). Immunoblots images were acquired using a LICOR (model).

Data analysis

LC-MS acquired spectra were analysed using XCalibur Qual Browser and XCalibur Quan Browser software (Thermo Fisher Scientific) by referencing to an internal library of compounds. Calibration curves were generated using synthetic standards of the indicated metabolites. PCR experiments were analysed using the ΔΔCt method. For RNA-Seq, reads were mapped to the mouse reference genome GRCm38 with the STAR (v.2.6.0c) aligner (Dobin, A. et al., 2013), filtered out using Cutadapt (version 1.10.0) (Martin, M., 2010), counted using the Bioconductor package Rsubread (v.1.28.1) (Liao, Y. et al., 2013) and gene annotated with GENCODE (release M17). Differential expression analysis was carried out with DESeq2 (v.1.18.1) (Love M. I. et al., 2014). Gene enrichment analysis was performed using the Gene Set Enrichment Analysis (GSEA) software from Broad Institute. Data compiling, processing and statistical analysis was performed using Microsoft Excel 2016 (v.16.16.27) and GraphPad Prism 7 (v.7.1.1) softwares.

For manuscripts utilizing custom algorithms or software that are central to the research but not yet described in published literature, software must be made available to editors and reviewers. We strongly encourage code deposition in a community repository (e.g. GitHub). See the Nature Portfolio guidelines for submitting code & software for further information.

## Data

Policy information about availability of data

All manuscripts must include a data availability statement. This statement should provide the following information, where applicable:

- Accession codes, unique identifiers, or web links for publicly available datasets
- A description of any restrictions on data availability
- For clinical datasets or third party data, please ensure that the statement adheres to our policy

All data are included within the article or the supplementary information. The source data for quantifications represented in all graphs plotted in figures and extended data figures are provided with this paper. Full versions of all gels and blots are provided in Supplementary Figures. Raw FastQ files for RNA-seq analyses are publicly available in the Gene Expression Omnibus (GEO) repository with the accession code GSE183745 (https://www.ncbi.nlm.nih.gov/geo/query/acc.cgi?acc=GSE183745).

## Human research participants

Policy information about studies involving human research participants and Sex and Gender in Research.

| | |
|---|---|
| Reporting on sex and gender | Findings apply to both genders and gender was not considered in the study design. Gender-specific data was not collected. Gender has no impact on the phenotype generated. |
| Population characteristics | Adult patients with suspected or confirmed inherited kidney cancer risk syndromes |
| Recruitment | patients were identified at the Specialist Multidisciplinary Team meeting and approached for informed consent at clinic consultations |
| Ethics oversight | Full ethics approval from West of Scotland Research Ethics Service, Dykebar Hospital, PAISLEY (REC reference: 16/WS/0039) |

Note that full information on the approval of the study protocol must also be provided in the manuscript.

# Field-specific reporting

Please select the one below that is the best fit for your research. If you are not sure, read the appropriate sections before making your selection.

☒ Life sciences ☐ Behavioural & social sciences ☐ Ecological, evolutionary & environmental sciences

For a reference copy of the document with all sections, see nature.com/documents/nr-reporting-summary-flat.pdf

# Life sciences study design

All studies must disclose on these points even when the disclosure is negative.

Sample size

For in vivo experiments, the number of animals was determined based on the number of animals implemented in previously published papers and determined to be adequate based on the magnitude and consistency of measurable differences between groups. For all in vitro assay, n=minimum 3 and up to 6 biological replicates were used for higher reliability and sufficient size for statistical analysis. Each biological replicate is defined as an independent culture of cells. The sample size is described in the relevant Figure legends and/or method section.
fig 1b,g n=7 min; fig 2a,b n=5; fig 2f,g,h,i,j,l,m,n ==3; fig 3b,c,d,e,f n=3; fig 3g,h,i n=4; fig 3k,p n=5; fig 3n n=9; fig 4d,e,g,h,j,k n=3; fig 4i,m n=4; fig 5b n=3; fig 5d n=5(N)/20(T); EDfig 2d,f n=4; EDfig 2e,f n=5; EDfig 2g,h,i,j,k,m,n,o n=3; EDfig 3 c,e,f,i,j,k,l,n n=3; EDfig 3h n=5; EDfig 4a,e,f,g,k n=3; EDfig 4h,i,j n=4; EDfig 5b,d,f n=3; EDfig 6b,c,d n=5; EDfig 6f,h n=3; EDfig 7c,d,g,i n=3; EDfig 7e,f n=5; EDfig 8b,c,d,e,f,g,h n=3; EDfig 8l n=5; EDfig 9a,c n=3; EDfig 9d,i n=5; EDfig 9e,f n=4; EDfig 9g n=9; EDfig 9l n=5; EDfig 10c,d,e,f n=3; EDfig 10i n=4; EDfig 11b,c,e,f,h,i,k,l,m,n,o,p,q n=3; EDfig 12d,f,g n=3 independent experiments.
For image quantification, experiments are presented from 3 independent experiments (otherwise specified) with a total number of cells (n)

analysed as: Fig. 2f-h: n= 173, 157, 158, 170, 160, 157, 162 for NT, vehicle, d1, d3, d6, d10, d15, respectively. Fig. 2i, j: n =30, 32, 35, 38, 32, 40, 36 and number of ROI analysed: n= 84, 85, 84, 85, 85, 84, 85 for NT, vehicle, d1, d3, d6, d10, d15, respectively. Fig. 3be, f: n=221, 192, 185 for vehicle, 200 µM MMF, 400 µM MMF, respectively. Fig. 4d, e: for TOM20-PDH+DNA+ vesicles analysis: n=166, 180, 167, 155, 170, for NT, 2d, 4d, 6d, 8d, respectively. Fig. 4e: for cytosolic DNA foci analysis: 165, 171, 162, 152, 160 for NT, 2d, 4d, 6d, 8d, respectively. Fig. 4g: n= 156; number of vesicles analysed: n=155. Fig.4h and Fig. ED12b: n= 408 ROI analysed. Fig. 4j, k: for TOM20-PDH+DNA+ vesicles analysis: n=158, 173, 158 for NT, MMF 6d si scramble, MMF 6 days si Snx9, respectively. Fig. 4k: for cytosolic DNA foci analysis: n=151, 166, 197, for NT, MMF 6d si scramble, MMF 6d si Snx9, respectively. Fig. ED2g-i: n= 166, 159, 166, 165, 175, 181, 170 for NT, vehicle, d1, d3, d6, d10, d15 respectively. Fig. ED2j, k: n =35, 42, 43, 47, 50, 48, 36 and number of ROI analysed: n= 99, 115, 137, 96, 127, 127, 76 for NT, vehicle, d1, d3, d6, d10, d15, respectively. Fig ED2l: number of mitochondria analysed: 11, 22, 79, 54, 46 for cFh1+/+, cFh1+/+ + Cre, cFh1fl/fl, cFh1-/-CL1, cFh1-/-CL19, respectively. Fig. ED3c, and h-i: n= 272, 246, 306, 300 for cFh1fl/fl, cFh1-/-CL1, cFh1-/-CL19, cFh1-/-CL1 + pFH-GFP. Fig. ED3e 30 cells were analysed per condition with a number of mitochondria analysed of n= 429, 827, 780 for cFh1fl/fl, cFh1-/-CL1, cFh1-/-CL19, respectively. Fig. ED3f: n= 33, 45, 67, 39 and number of ROI analysed: n= 79, 71, 164, 73 for cFh1fl/fl, cFh1-/-CL1, cFh1-/-CL19, cFh1-/-CL1 + pFH-GFP, respectively. Fig. ED4e-g: n=163, 161, 163 for Sdhbfl/fl, Sdhb-/-CL5, Sdhb-/-CL7, respectively. Fig. ED5d: n= 174, 165, 171 for cFh1fl/fl, cFh1-/-CL1 +pEGFP, cFh1-/-CL1 +pEGFP:NDI1, respectively. Fig. ED5f: n=221, 192, 185 for vehicle, 200µM MMF, 400µM MMF, respectively. Fig. ED6h: n= 169, 188 for DMSO and MMF 8d, respectively. Fig. ED7g: n= 182, 156, 189, 228 for cFh1fl/fl, cFh1-/-CL1, cFh1-/-CL1+pcytoFH-GFP, cFh1-/-CL1+pFH-GFP respectively. Fig. ED8b-d: n=152, 153, 162, 155, 155 for vehicle, DMS 200 µM, DMS 400µM, DMS 1mM, DMS 5mM, respectively. Fig. ED9c: n= 933, 660 for DMSO and MMF8d , respectively. Fig. ED10c: n= 175, 171, 183, 168, 166, 187, 188, 186, 154, 184, 161, 180, 169, for cFh1fl/fl, cFh1-/-CL1 si scramble, cFh1-/-CL1 si Snx9, cFh1-/-CL1 si Vdac1, cFh1-/-CL1 si Bax/Bak, cFh1-/-CL1 si Rab9, cFh1-/-CL1 si Drp1, cFh1-/-CL1 si Opa1, cFh1-/-CL1 si Mfn1, cFh1-/-CL1 si Mfn2, cFh1-/-CL 1si cGas, Fh1-/- si Sting, cFh1-/-CL1si Rig-I, respectively. Fig. ED10g: n=31, 32, for cFh1fl/fl and cFh1-/-CL1, respectively. Fig. ED11b-c: n=151, 154, 166, 159, 161, 163, for cFh1fl/fl, cFh1fl/fl si scramble, cFh1fl/fl si Snx9, cFh1-/-CL1 si scramble, cFh1-/-CL1 si Snx9, cFh1-/-CL19si scramble, cFh1-/-CL19si Snx9, respectively. Fig. ED11e, f: n= 55, 50, 53, 55, 60, 53 and number of ROI analyzed: n= 168, 162, 158, 348, 125, 155, 76 for cFh1fl/fl+ si scbl, cFh1fl/fl + si snx9, cFh1-/-CL1+si scbl, cFh1-/-CL1+ si snx9, cFh1-/-CL19+ si scbl, cFh1-/-CL19 + si snx9, respectively. Fig. ED11h, i: n=191, 167, 160, 179. 190, 147, 160, for vehicle, d6 si scramble, d6 si Snx9, d10 si scramble, d10 si Snx9, d15 si scramble, d15 si Snx9, respectively. Fig. ED11n-p: n=166, 180, 167, 155, 170, for NT, 2d, 4d, 6d, 8d MMF, respectively. Fig. ED11q: n=151, 166, 197 for NT, MMF 6d si scramble, MMF 6d si Snx9, respectively. Fig. ED12a: n=85. Fig. ED12d: n= 189, 155, 272, 159, 159, 144 for vehicle, 4OHT d1, d3, d6, d10, d15, respectively. Fig. ED12f: n= 170, 167, 145, 136, 133, 136, 139 for Fh1+/++DMSO, Fh1+/+ + MMF 6d, Fh1+/+r0+ DMSO Fh1+/+r0 + MMF 1d, 3d, 6d, 8d, respectively.

Data exclusions

No data from in vivo samples were excluded from analysis but samples from some animals where the treatment had to be interrupted due to health concern were not collected.
In Extended Data Fig. 2c and Extended Data Fig. 6i, up to 2 biological replicates (out of 5 in total) per sample were excluded from all analyses due to bad sample quality/poor yield (data not shown).

Replication

In vivo and in vitro experiments were performed with at least 7 or a minimum of 3 (up to 6 times) biological replicates, respectively. All attempts at replication gave similar results and results were reliably reproduced with the same trend.

Randomization

Age-matched mice were randomly allocated into experimental groups.
For imaging, the cells and sample regions were evenly allocated and selected randomly. There was no requirement for randomization of other data.

Blinding

As no subjective measurements were done and the analysis were performed with quantitative instruments, no blinding was performed.

# Reporting for specific materials, systems and methods

We require information from authors about some types of materials, experimental systems and methods used in many studies. Here, indicate whether each material, system or method listed is relevant to your study. If you are not sure if a list item applies to your research, read the appropriate section before selecting a response.

## Materials & experimental systems

| n/a | Involved in the study | |
|---|---|---|
| ☐ | ☒ | Antibodies |
| ☐ | ☒ | Eukaryotic cell lines |
| ☒ | ☐ | Palaeontology and archaeology |
| ☐ | ☒ | Animals and other organisms |
| ☒ | ☐ | Clinical data |
| ☒ | ☐ | Dual use research of concern |

## Methods

| n/a | Involved in the study | |
|---|---|---|
| ☒ | ☐ | ChIP-seq |
| ☒ | ☐ | Flow cytometry |
| ☒ | ☐ | MRI-based neuroimaging |

## Antibodies

Antibodies used

Immunobloting
goat polyclonal anti-FH/Fumarase (AbCam, ab113963) and anti-beta Actin [AC-15] (AbCam, ab6276), rabbit polyclonal anti-Grp75 (AbCam, ab2799), anti-Mfn1 (AbCam, ab126575), anti-P-IRF3S386 (AbCam, ab76493), and mouse monoclonal anti-VDAC1 (AbCam, ab14734). Mouse monoclonal anti-Actin (Sigma-Aldrich, A2228) and anti-Vinculin (Sigma-Aldrich, V4505). Mouse monoclonal anti-Drp1 (BD Transduction Laboratories, 611113), and anti-Opa1 (BD Transduction Laboratories, 612607). Rabbit polyclonal anti-Mfn2 (Cell Signaling Technology, 11925), anti-Bak (Cell Signaling Technology, 12105), anti-Bax (Cell Signaling Technology, 2772), anti-cGas (Cell Signaling Technology, 31659S), anti-Irf3 (Cell Signaling Technology, 4302S), anti-Stat1 (Cell Signaling Technology, 9172S), anti-P-Stat1Tyr701 (Cell Signaling Technology, 9167S), anti-TBK1/NAK (Cell Signaling Technology, 3013S), anti-Phospho-TBK1/NAKSer172 (Cell Signaling Technology, 5483S), anti-Rab9A (Cell Signaling Technology, 5118S), anti-Rig-1(Cell Signaling Technology, 3743S), and

| | |
|---|---|
| | anti-Sting (Cell Signaling Technology, 50494S). Rabbit polyclonal anti-Snx9 (Proteintech, 15721-1-AP). Mouse monoclonal anti-Cytochrome c (BD Pharmingen, 556433). Rabbit polyclonal anti-mtTFAM (GeneTex , GTX103231).<br><br>Immunofluorescence<br>mouse monoclonal anti-DNA (Millipore, CBL186). Rabbit polyclonal anti-TOM20 (AbCam, ab232589), mouse monoclonal anti-TOM20 (AbCam, ab56783), and anti-PDH (AbCam, ab110333), were purchased from Abcam. Anti-Cytochrome c (BD Pharmingen, 556432). Donkey anti-mouse, goat anti-mouse IgG1, goat anti-mouse IgG2a, goat anti-mouse IgGM, and goat anti-rabbit Alexa Fluor 488, 565, 594 or 647 were used as secondary antibodies (all from Invitrogen). Rabbit anti-Cgas (Cell Signalling, D3080). Mouse anti-GM130 (BD BioSciences, 610822). |
| Validation | All antibodies from commercial vendors were validated by the manufacturers on their websites. |

# Eukaryotic cell lines

Policy information about cell lines and Sex and Gender in Research

| | |
|---|---|
| Cell line source(s) | Cell lines source(s)<br>All cell lines were isolated from transgenic embryonic mouse kidneys. The constitutive cell lines cFh1FL/FL, cFh1-/- clones 1 and 19 are described in Frezza et al. DOI: 10.1038/nature10363. The constitutive cell line cFh1-/- clone 19 + pFH are described in Sciacovelli et al. https://doi.org/10.1038/nature19353. The inducible cell lines iFh1 clones 29 and 33 were isolated using the protocol described in Mathew at al. DOI: 10.1016/S0076-6879(08)01605-4. |
| Authentication | The cell lines were not authenticated. |
| Mycoplasma contamination | All used cell lines were routinely tested and confirmed negative for mycoplasma contamination. |
| Commonly misidentified lines<br>(See ICLAC register) | No commonly misidentified cell lines were used. |

# Animals and other research organisms

Policy information about studies involving animals; ARRIVE guidelines recommended for reporting animal research, and Sex and Gender in Research

| | |
|---|---|
| Laboratory animals | Mice were of mixed genetic background C57BL/6 and 129/SvJ. Animals were bred and maintained under specific pathogen-free conditions at the Breeding Unit (BRU) at the CRUK Cambridge Institute (Cambridge, UK). Fh1fl/fl and R26Creert2 mice were gifts from Prof Gottlieb (Technion, Israel Institute of Technology, Israel) and Dr Winton (CRUK, Cambridge Institute, Cambridge, UK), respectively. Experimental mice were homozygous for the conditional LoxP-exon3/4-LoxP Fh1 allele and expressed the Cre–recombinase-ert2 fusion under control of the ROSA26 promoter (Fh1fl/fl; R26 Creert2/Creert2). Littermate controls lacked the LoxP-exon3/4-LoxP allele but also expressed the Cre-ert2 allele under the control of the ROSA26 promoter (Fh1+/+; R26 Creert2/Creert2). Control mice were induced and sacrificed at the same time as their experimental littermates. In vivo experiments (tamoxifen induction) were performed under specific pathogen-free conditions at the Breeding Unit (BRU) at the CRUK Cambridge Institute (Cambridge, UK). All mouse experiments were performed in individually ventilated cages under the Animals (Scientific Procedures) Act 1986 (project licence P8A516814). The experiments were not randomised, and investigators were not blinded to treatment status during experiments and outcome assessment. |
| Wild animals | *Provide details on animals observed in or captured in the field; report species and age where possible. Describe how animals were caught and transported and what happened to captive animals after the study (if killed, explain why and describe method; if released, say where and when) OR state that the study did not involve wild animals.* |
| Reporting on sex | Findings apply to both genders and animal gender was not considered in the study design. Male and female animals were randomly assigned to study cohorts. Gender-specific data was not collected. Gender of the animals has no impact on the phenotype generated. |
| Field-collected samples | *For laboratory work with field-collected samples, describe all relevant parameters such as housing, maintenance, temperature, photoperiod and end-of-experiment protocol OR state that the study did not involve samples collected from the field.* |
| Ethics oversight | The Project Licence has been considered and granted by the UK Home Office; and ethically approved by the local establishment (LMB AWERB). |

Note that full information on the approval of the study protocol must also be provided in the manuscript.
